# Human Enterovirus D68 infection – the intricate dance of cells, genes, and invading bugs

**DOI:** 10.3389/fcimb.2025.1667268

**Published:** 2025-11-07

**Authors:** Hanne Lillerovde Ørstenvik, Ann-Kristin Tveten, Yanran Cao

**Affiliations:** Department of Biological Sciences Ålesund, Faculty of Natural Sciences, Norwegian University of Science and Technology (NTNU), Ålesund, Norway

**Keywords:** human enterovirus D68, respiratory epithelium, streptococcus pneumoniae, streptococcus pyogenes, respiratory infection, co-infection

## Abstract

The respiratory tract is particularly vulnerable to infections from various pathogens, often leading to severe illnesses. Co-infections involving multiple pathogens are commonly observed in respiratory diseases, although their underlying mechanisms remain poorly understood. Lung epithelial cells play a crucial role in the body’s defense and are primary targets for many pathogens, which exploit them for attachment and entry. This study investigates the molecular mechanism underlying co-infections of human enterovirus D68 (HEV-D68) and bacteria (Group *A Streptococcus* and *Streptococcus pneumoniae*) in lung epithelial cells. Cell viability and gene expression changes were assessed over a 24-hour period. The results revealed significant cytopathic effect and distinct gene expression patterns. HEV-D68 infection alone induced stronger upregulation of mucin genes (*MUC2*, *MUC5AC*) and immune markers (*TNFα* and *p38*) compared to co-infections. In contrast, co-infections led to downregulation of sialic acid biosynthesis genes (*CMAS*, *GNE*, *NANS*), suggesting impaired receptor restoration and altered host-pathogen dynamics. These findings contribute to a deeper understanding of epithelial responses and highlight potential therapeutic targets.

## Introduction

The respiratory tract is highly susceptible to infections caused by various pathogens, often resulting in severe illnesses. While infections typically involve a single pathogen, co-infections with multiple pathogens are also common (Oliva and Terrier, 2021). The mechanisms underlying viral-bacterial co-infections are not yet fully understood, although both types of pathogens use similar invasion strategies. Host cells initiate programmed cell death to limit infection, but pathogens can evade these defenses by mimicking host molecules or releasing proteins that inhibit immune responses (Ashida et al., 2011; Thakur et al., 2019). Ongoing research aims to better understand these pathogen-host interactions to gain insights into how cells are affected and how pathogens overcome defenses.

Epithelial cells in the lungs play a crucial role in the body’s defense mechanisms and are primary targets for respiratory pathogens (Burgoyne et al., 2021). These cells express surface molecules, such as proteins and glycoproteins, which are essential for cellular communication and function. Many pathogens exploit these molecules for attachment and entry. Viruses use them as receptors to bind to and enter host cells, hijacking the cell’s machinery to replicate - often resulting in cell death. Bacteria, on the other hand, may use these surface molecules as nutrients or receptors, breaking them down for energy to grow and reproduce (Indraratna et al., 2022; Jennings et al., 2022; Rai et al., 2016). Understanding these interactions is key to developing effective strategies for preventing and treating infections.

Pathogen invasion activates the innate immune system, beginning with pathogen recognition through toll-like receptors (TLRs) and activation of signaling pathways. This can lead to a cytokine storm to prevent viral entry (El-Zayat et al., 2019). Viral infections can disrupt the epithelial barrier, causing cell death and facilitating secondary bacterial infections. This disruption enables pathogens to spread within the host, potentially leading to severe disease. Some pathogens exploit the same structural molecules in the respiratory tract to invade host cells.

Glycolipids and sialic acid play a central role in pathogen-cell interactions (Haines-Menges et al., 2015; Lewis et al., 2022). Gangliosides, a type of glycolipid, consist of carbohydrate oligosaccharides with one or more sialic acid residues attached (Traving and Schauer, 1998). Sialic acid is a type of negatively charged sugar naturally present in the membranes of all cells and is essential for mucus formation in the respiratory epithelium (Varki, 2008; Zhu et al., 2024). One of the most common forms of sialic acid in humans is N-acetylneuraminic acid (Neu5Ac). Sialic acid is a common carbohydrate that plays a key role in viral entry into host cells, serving as a receptor for various viruses. It is also exploited by a diverse group of bacterial pathogens (Jennings et al., 2022).

**Figure 1:**
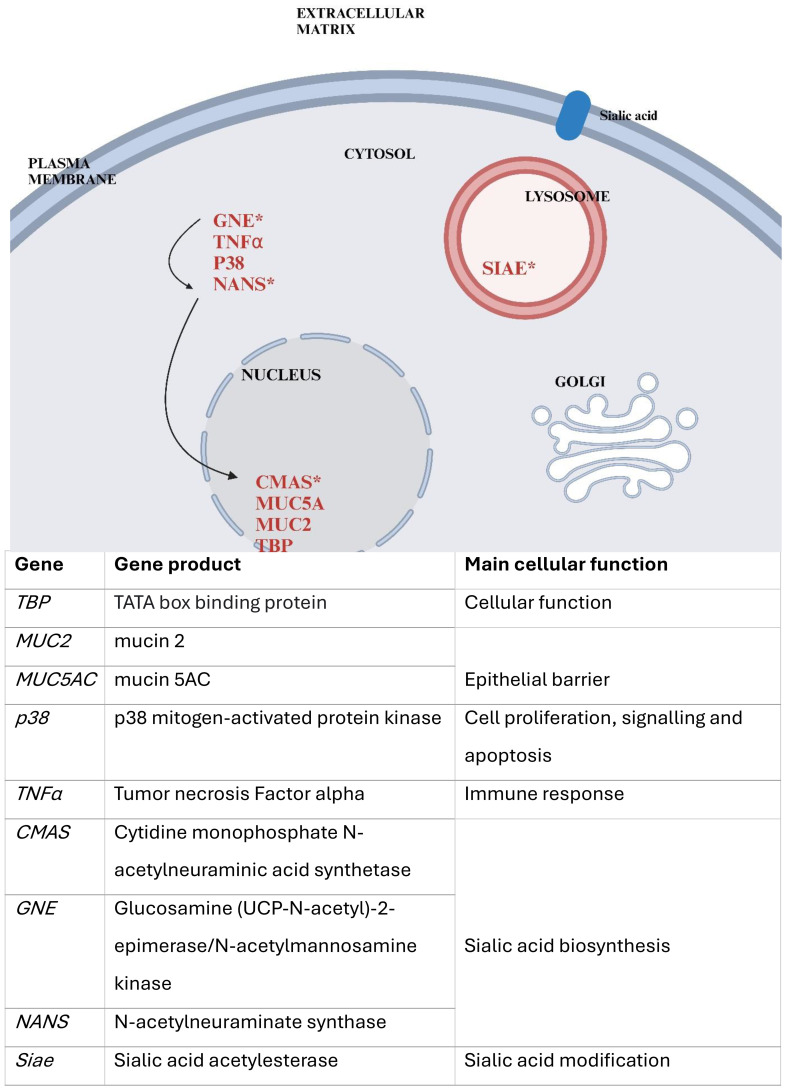
Provides a simplified overview of the cellular localization of the selected gene products and their roles. Genes tested in this study are marked in red, those involved in sialic acid biosynthesis indicated by an asterisk (*). Arrows represent the biosynthetic pathway of sialic acid, beginning with GNE, followed by NANS, and later CMAS. The figure was created using Biorender and is based on the figure in NCBI: Sialic Acids - Essentials of Glycobiology^(X)^.

In the present study, we investigated the gene expression of lung epithelial cells infected with human enterovirus D68 (HEV-D68), as well as co-infections with Group *A Streptococcus* (GAS) and *Streptococcus pneumoniae*. Biomarkers related to cellular responses during infection and sialic acid biosynthesis were selected, as illustrated in **Figure 1** below. A panel of nine biomarkers was chosen, encompassing functions such as epithelial barrier integrity, immune response, and sialic acid metabolism. These biomarkers were selected to enhance our understanding of the cellular response and sialic acid synthesis during viral and bacterial co-infection, where all pathogens utilize sialic acid.

Human enterovirus D68 (HEV-D68) causes respiratory infections through receptor-mediated endocytosis and shares characteristics with both rhinovirus and enterovirus. HEV-D68 binds to specific receptors on the host cell surface, with some strains recognizing sialic acid. The virus anchors its proteins to sialic acid at a conserved site formed by the viral proteins VP1 and VP3 (Liu et al., 2015), with specific amino acid residues facilitating this interaction. Strain-specific receptor preferences have been observed; while some strains use sialic acid for entry, others rely on alternative receptors (Sooksawasdi Na Ayudhya et al., 2021). HEV-D68 is increasingly recognized as a significant respiratory pathogen due to its role in respiratory infections and potential for co-infection with bacteria and other viruses (Foster et al., 2015).

HEV-D68 can affect epithelial cells, including A549 cells, increasing their susceptibility to secondary bacterial infections through mechanisms such as cell death, disruption of tight junctions, and increased permeability. Infection may also lead to elevated mucus production, obstructing airways and reducing pulmonary capacity, thereby contributing to respiratory distress. The immune response to HEV-D68, characterized by the release of cytokines and chemokines, exacerbates inflammation and mucus production (Naeem et al., 2025). The interaction between *S. pneumoniae* and the immune system can either suppress or overstimulate immune responses, influencing viral replication and spread.

Respiratory infections caused by various viruses can lead to secondary bacterial infections, which often increase the severity of the initial viral infection. Bacteria such as *Streptococcus pneumoniae* and *Streptococcus pyogenes* (Group A Streptococcus - GAS) exploit sialic acid as an energy source. These bacteria are commonly found in the respiratory tract and can cause a range of infections, from mild to severe. Both species possess enzymes that metabolize sialic acid residues, thereby exposing underlying receptors that facilitate bacterial invasion (Ryan et al., 2001; Sharapova et al., 2018). Unlike HEV-D68, which binds to sialic acid as a receptor for cell entry, these bacteria utilize sialic acid for energy metabolism. We hypothesize that co-infection with HEV-D68 and either GAS or *S. pneumoniae* may induce distinct gene expression changes in the epithelial cells compared to the viral mono-infection.

## Aim

This study aims to investigate the molecular mechanisms underlying co-infections involving HEV-D68 and bacterial pathogens in lung epithelial cells. We conducted an *In vitro* experiment by infecting A549 lung epithelial cells with human enterovirus D68 (HEV-D68) and two different bacterial species: GAS and *S. pneumoniae.* The goal is to better understand the pathogen-host cell interactions, with a particular focus on the cellular response and the biosynthesis of sialic acid exploited by pathogens for replication and spread.

## Materials and Methods

### *In vitro* cell culture

Human lung epithelial (A549) cells were obtained from the American Type Culture Collection (ATCC, CCL-185). Cells were cultured in Kaighn’s modification of Ham’s F-12 medium (F-12K medium), supplemented with 10% fetal bovine serum (FBS; Gibco, Thermofisher, US) and 1% Antimycotic Antibiotics 100X (Gibco, Thermofisher, US). Cultures were maintained at pH 7.42 at 37°C, and 5% CO_2_ in a humidified incubator. Cells were grown in ventilated tissue culture flasks and maintained in growth medium until use.

Bacterial species. Both Group A streptococcus (*Streptococcus pyogenes Rosenbach*, ATCC – 19615) and *S. pneumoniae* (*Streptococcus pneumonia (Klein) Chester*, ATCC – 49619) were obtained from ATCC. Both bacteria strains were inoculated on blood agar plates and incubated at 37°C and 5% CO_2_ humidity. Bacterial growth was monitored microscopically to ensure viability and purity.

Human Enterovirus D68 (HEV-D68) strain US/IL/14-18952 (VR-1824™) with taxonomy ID: 167331, was obtained from the ATCC (Manassas, VA). The virus was propagated in A549 cells at 33°C, following protocols described by Elrick et al. (2021) (Elrick et al., 2021). The viral growth medium consisted of DMEM/F-12 medium supplemented with 10% FBS. Cytopathic effect (CPE), characterized by rounded and refractile cells forming clusters across cell layers, were observed four days past infection (DPI). Viral titers were determined, and samples were aliquoted and stored at –80°C. An overview of the cell lines, bacterial strains, and viral propagation conditions used in this study is provided in **Table 1**. 

**Table 1:** Summarizes the biological materials used in this study, including cell lines, virus, and bacterial strains, along with their abbreviations, source and catalog numbers.

Type	Name	Abbreviation	Strain	Source	Cat. No
*Cell line*	Lung epithelial cells	A549		ATCC	CCL-185
*Virus*	Human Enterovirus D68	HEV-D68	US/IL/14-18952	ATCC	VR-1824
*Bacteria*	Streptococcus pneumoniae	*S. pneumoniae*		ATCC	49619
	Group A *Streptococcus*	GAS		ATCC	19615

### Viral titration

To determine the infectious dose of HEV.D68, a TCID_50_ assay was performed. Serial dilutions of the virus ranging from 10^0^ to10^-8^, were prepared, and 200 μl from each dilution was added to wells in 96-well plates containing A549 cells. Plates were incubated under optimal conditions until the CPE was observed. After two days, the medium was removed, and the wells were washed, fixed with methanol, and stained with 0.1% crystal violet. Absorbance was measured at 570 nm using an ELISA plate reader. TCID_50_ value were calculated using the Reed and Muench method (REED and MUENCH, 1938), based on the amount of cell lysis across dilutions. The TCID_50_ was determined to be 10^-3^.

### Experimental design

#### Cell counts and cell preparations

A549 alveolar type 2 lung epithelial cells were used to evaluate HEV-D68 infection and bacterial co-infection. Cells were cultured in 75cm^2^ cell flasks (ref. *734-2313*, VWR), and detached using 0.25% Trypsin-EDTA for 10 minutes. For infection experiments, cells were seeded at a density of 300–000 cells/ml in 6-well plates (*ref. 734-2323*, VWR) one day prior to infection. A total of 18 plates were prepared.

For MTT assay A549 cells were seeded in 96-well plates at a density of 5 x 10^4^ cells/well and placed in incubator to allow the cells to adhere for 24 hours. 10-fold dilution of both bacterial species (10^0^ to 10^-8^) was added to each row in the 96-well plate and incubated for 6 hours, including control with untreated cells. After bacterial treatment, 10 μl of MTT solution was added to each well and incubated for 4 hours at 37°C. The medium was removed, and 100 μl of DMSO was added. Absorbance was measured at 570 nm using an ELISA plate reader. The assay was performed according to the manufacturer’s instructions using the Cell proliferation Kit 1 (MTT) (Roche, Cat.no. 11465007001; Sigma-Aldrich)(Diagnostics).

#### Co-infection of lung epithelial cells

All samples were prepared in biological duplicates. For each time point, one 6-well plate was used per condition. The four experimental conditions included; control (uninfected A549 cells), HEV-D68 (strain US/IL/14-18952, VR-1824), GAS (ATCC 19615) mixed with HEV-D68, and *S. pneumoniae* (ATCC 49619) mixed with HEV-D68, respectively as listed in **Table 2**. Prior to infection, the growth medium was removed and cells were washed with 3 ml Dulbecco’s phosphate buffered saline (DPBS; Cat.no *RNBH5435, Sigma*). Pathogens were added to 3 ml fresh growth medium, with final concentrations of 4xTCID_50_ for the virus and 10^8^ CFU/ml for bacteria. Plates were incubated for 6 hours at 37°C and 5% CO_2_.

**Table 2:** Experimental conditions and sampling schedule for co-infection with HEV-D68 (VR-1824), Streptococcus pneumonia (ATCC 49619) and GAS (ATCC 19615).

Experimental conditions	Cells	Infection	Sampling
Control	A459		T6, T12, T24
*HEV-D68 (VR-1824)*	A549	6 hours	T6, T12, T24
*HEV-D68 +* GAS *(ATCC 19615)*	A549	6 hours	T6, T12, T24
*HEV-D68 + S. pneumoniae* (*ATCC 49619)*	A549	6 hours	T6, T12, T24

After 6 hours, the medium was removed and cells were washed with 2 ml DPBS. Fresh growth medium was added, and samples were collected at 6 (T6), 12 (T12), and 24 (T24) hours post-infection using a cell scraper (*Cat.no 734-2602*, VWR) and RLT buffer (*Cat.no 74106*, Qiagen). Samples were stored directly at -80°C.

### Quantitative real-time PCR

#### RNA extraction

Cells were harvested at four different time points within the first day post-infection (1 DPI). After removing the medium, 500 μl RLT buffer was added to each well. Cells were detached using a cell scraper and transferred to 2 ml Eppendorf tubes. RNA was extracted using the RNeasy Plus Mini Kit (Cat.no *74106*, Qiagen), excluded the use of G-columns to preserve RNA integrity. Samples were stored at -80°C. RNA quality was assessed using the Qubit RNA IQ Assay kit (*Cat.no Q33221*, Invitrogen), on randomly selected samples (data not shown).

#### RT-qPCR – gene expression analysis

RNA was reverse transcribed into cDNA using the qScript cDNA synthesis kit (*cat.no 95047*, Quantabio). Reactions were prepared in 20 μl volumes, containing 10 μl RNA, 4 μl reaction mix, and 1 μl enzyme. Tubes were placed in the thermocycler (*2720 Thermal Cycler, Applied Biosystems*) and cDNA synthesis was administered the following program: one cycle for 5 min at 22°C, one cycle for 30 min at 42°C and one cycle at 85°C for 5 min, inclosing 4°C. cDNA concentrations were measured using Qubit 4.0 (*Q3326*, Invitrogen) and the Qubit 1X dsDNA HS assay kit (*Q33230*, Invitrogen), ensuring normalization to 400ng/μl for each PCR reaction.

For each sample, nine different genes of interest were analyzed in silico, whereas TBP are housekeeping gene used as an endogenous control. Primer sequences and geneID are listed in **Table 3** below.

**Table 3:** Shows the biomarkers included in this study, their assigned GeneID^(Y^ and the associated primer sequences for each gene.

Gene	Gene ID	Sequence (5`- 3`)
*TBP*	6908	5′-CACGAACCACGGCACTGATT-3′ 5′-TTTTCTTGCTGCCAGTCTGGAC-3′
*MUC2*	4583	5′-TGGCTGGATTCTGGAAAACC-3′ 5′-GATACATGGTGGCTCTGCAA-3′
*MUC5AC*	4586	5′-GTGCTGTGTACCATAGGAGC-3′ 5′-CGAGCGAGTACATGGAAGAG-3′
*p38*	1432	5′-CGAGCTGTTGACTGGAAGAA-3′ 5′-TGGCTTGGCATCCTGTTAAT-3′
*TNFα*	7124	5′-AGTCTGGGCAGGTCTACTTT-3′ 5′-TCGAAGTGGTGGTCTTGTTG-3′
*CMAS*	55907	5′-GAGACGCCATCAGTTTCGAT-3′ 5′-CCCTGCAAGTAACCCATCTC-3′
*GNE*	10020	5′-TTCGTGGCGCTTGGTTC-3′ 5′-CAAGTAGCAACACAAACCCG-3′
*NANS*	54187	5′-GTGCTTCATCATTGCCGAG-3′ 5′-AGGAATGCTTCGAGGTGTATG-3′
*Siae*	54414	5′-CCGACAGAAGTGCAGGTATT-3′ 5′-TGAGCTTTCACACTGGTCAC-3′

Quantitative PCR was performed using Powerup SYBR Green master mix (Applied biosystems, *ref. A25742*), following the manufacturer’s instructions. Each reaction consisted of; 10 μl Power up SYBR green, 1.33 μl 500 nM primer and 400 pg/μl cDNA were pipetted in 96-MicroAmp plates (0.1 ml Applied Biosystems) sealed with adherent film (Microamp Optical Adhesive Film, Applied Biosystems) to secure the reaction volume. The final reaction volume per well was 20 μl. Plates were briefly centrifuged using a plate spin II centrifuge (Thermo Fisher Scientific) for 40 seconds at 3400 rmp before being loaded into the AriaMx Real-Time PCR system (Agilent Technologies), operated via its dedicated software.

Samples were analyzed using ROX as a passive reference dye. The qPCR program followed a three-step cycling protocol starting with an initial denaturation step of 95°C for 3 minutes. A total of 38 cycles of denaturation for 15 s at 95°C, annealing for 20 s at 58°C and extension for 30 s at 72°C as described by Hoem, K.S., Tveten, AK (Hoem and Tveten, 2023). Melt curve analysis was performed post-amplification to confirm primer specificity and assess amplification efficiency. Primer efficiency was validated using a ten-fold dilution series in duplicate. Technical duplicates were included for each sample. A difference of one Ct value between duplicates or a standard deviation (SD) > 0,5 for melting temperature (Tm) was considered unreliable, and the sample was rerun.

#### Data analysis and statistics

Raw gene expression data were exported from AriaMx software and processed in Microsoft Excel. Outliers were identified using Grubb’s test (GraphPad). Relative gene expression was calculated using the comparative 2^-ΔΔCt^ method of Livak and Schmittgen (Livak and Schmittgen, 2001). All qPCR reactions were performed in both biological and technical duplicates. Ct values from infected samples were compared to those from uninfected control cells. Transcriptional levels were normalized using a housekeeping gene. Initially, *GAPDH* and *TBP* were evaluated for stability using Genorm. *TBP* was identified as the most stable and therefore used as the reference gene for all calculations. Primer specificity was confirmed via melt curve analysis. Primer efficiency was calculated using the formula: efficiency (%) = (-1/10^slope^-1) × 100.

An unpaired t-test was used to compare gene expression between control and infected groups. All statistical analyses assumed normal distribution of data. Significance was determined based on ΔCt values, with p-values < 0.05 considered statistically significant. Statistical analysis was performed using IBM SPSS Statistics 29 (version 30.0.0.0, build 172). Figures were generated using GraphPad Prism (version10.3.1, build 509).

## Results

### Cell viability

**Figure 2:**
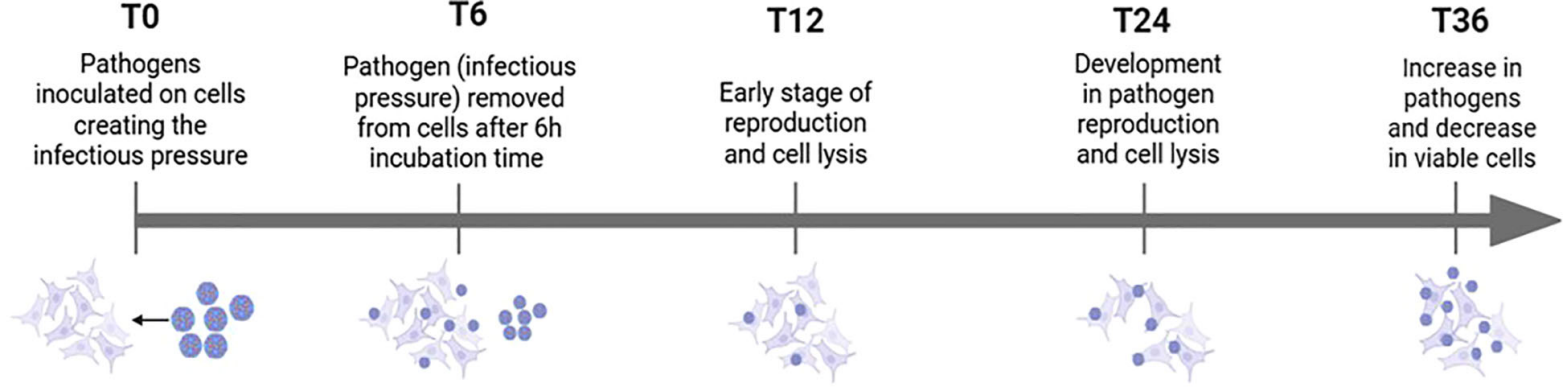
Overview of how infectious pressure develops over time, including pathogen-cell interactions and morphological changes observed under the microscope.

To ensure consistency across experimental conditions, cell viability was monitored 0-, 6-, 12- and 24-hours post-infection. The infectious pressure was evident from the beginning of the experiment and peak around the 6-hour time point. After sample collection at 6 hours (T6), the medium was refreshed to reduce the infectious load. However, infection continued due to pathogens already attached to the epithelial cells at the T6. **Figure 2** illustrates the progression of infectious pressure over time, based on morphological changes observed under the microscope. These changes, indicative of CPE, were used to assess the impact of infection. A549 lung epithelial cells typically display a spindle-shaped morphology, forming interconnected networks. Cells were infected at approximately 70% confluence to ensure optimal growth and responsiveness.

HEV-D68 infection resulted in visible CPE, with cells becoming rounded and detaching from the culture surface. At 70-80% confluence, A549 cells are in exponential growth phase, which coincides with active viral replication. By adding the virus at this confluence level, continuous cell splitting occurs parallel to increased CPE as the virus spread through the culture. Continuous monitoring of cell morphology and confluence provided valuable insight into the progression of viral infection and its impact on cell viability.

**Figure 3:**
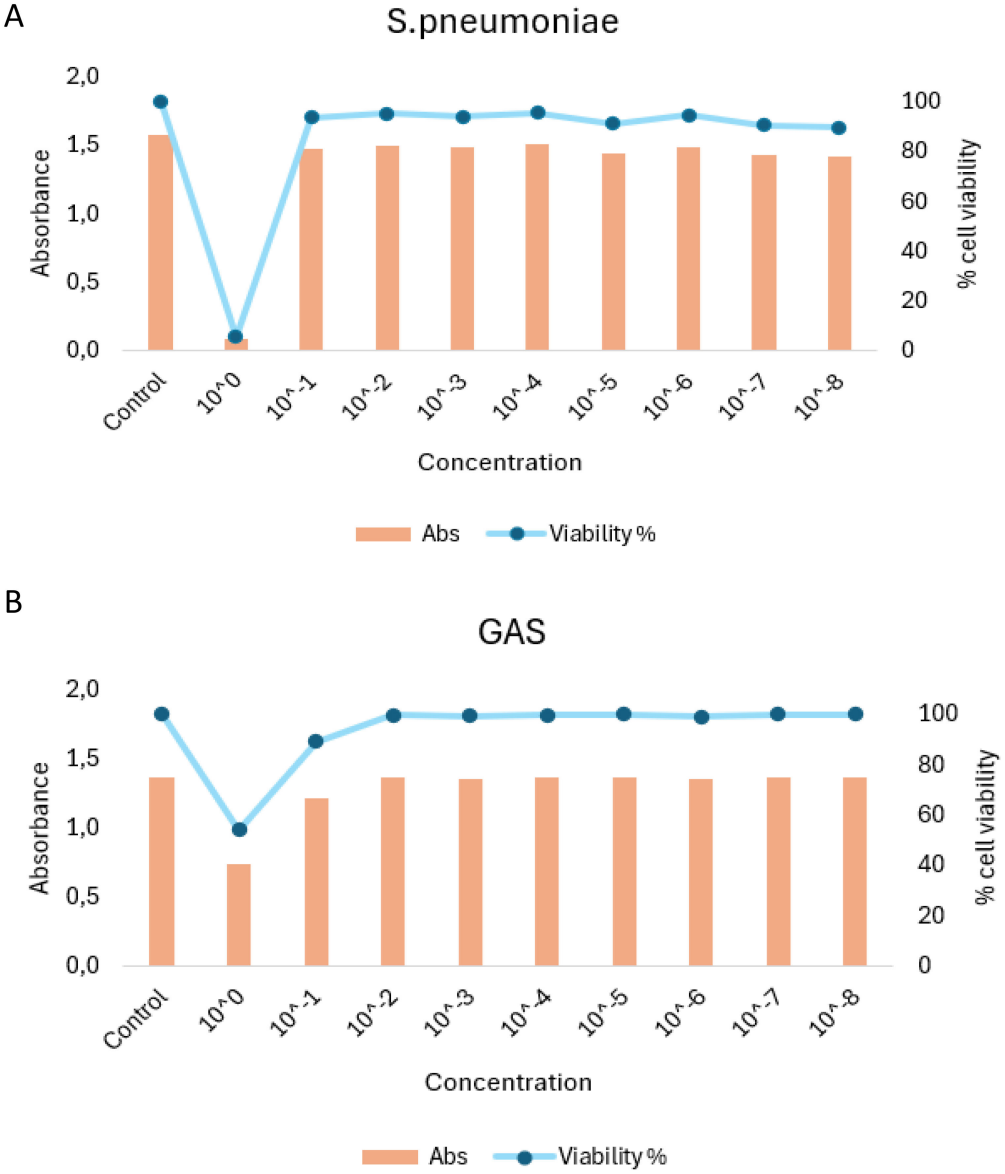
MTT viability assay results for A549 cells infection with S. penumoniae **(A)** and GAS **(B)**. Cells were exposed to infection pressure for 6 hours, followed by washing and incubation. Absorbance (Abs) values are shown on the left y-axis, percentage of visible cells on the right y-axis, and pathogen concentration on the x-axis.

Due to the increased CPE and lytic effects caused by both HEV-D68 and *S. pneumoniae*, the number of viable cells available for RNA isolation decreased significantly after 24 hours. Therefore, the experimental endpoint was set at 24 hours. To further assess cell viability, an MTT assay was performed after 24 hours (1 DPI) in A549 cells infected with *S. pneumoniae* and GAS. This assay confirmed the viability of cells and the ability of both bacterial strains to grow on A549 cells as showed in **Figure 3**.

Throughout the experiment, an increase in CPE was observed between 12 and 24 hours in both virus-only and co-infected conditions. All samples were visually inspected under an optical microscope prior to harvesting to confirm cell viability. Quality control was also performed before the experiment began. After 36 hours, pronounced differences in CPE were observed, reinforcing the decision to set the experimental cutoff at 24 hours. For future experiment requiring longer durations, the initial infection pressure would need to be reduced to maintain cell viability over extended periods.

### The cellular response to pathogen infection

Mucin genes play an important role in protecting epithelial cells from external threats (Mettelman et al., 2022). Mucins are classified into membrane-associated and secreted types. Secreted mucins, such as *MUC2* and *MUC5AC*, are highly glycosylated proteins that form a physical barrier to protect cells (Hollingsworth and Swanson, 2004). Investigating these genes provides insight into pathogenesis and host defense mechanisms. *TNFα* is a key component of the primary immune response to viral infections, promoting cytokine production and contributing to inflammation (Newton et al., 2016). The MAP kinase family, including p38, is involved in cellular processes such as growth, differentiation, and apoptosis (Hollingsworth and Swanson, 2004; Ono and Han, 2000). *p38* is activated by extracellular stimuli, making it a relevant marker for studying cellular stress responses (Banerji and Saroj, 2021).

**Figure 4:**
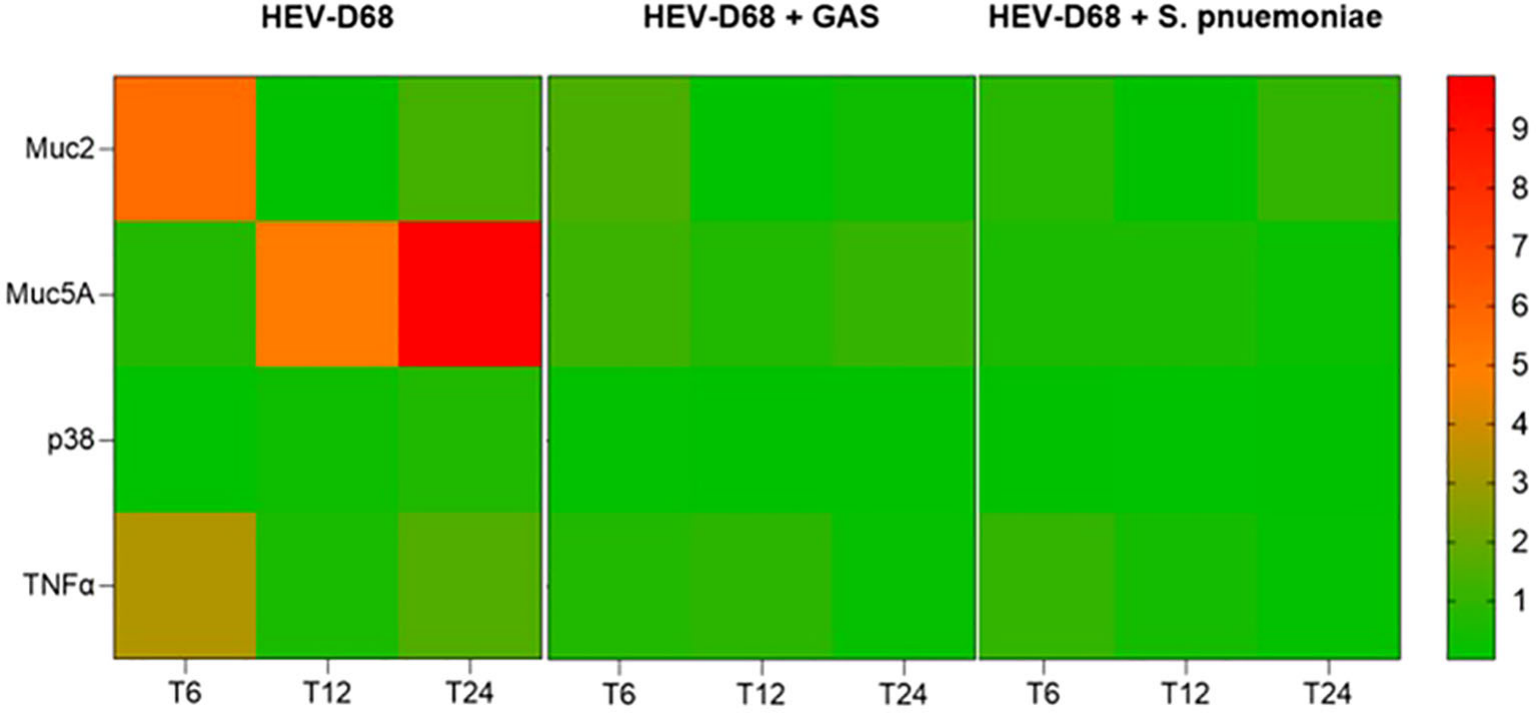
Presents a heatmap showing the relative expression of MUC2, MUC5AC, p38 and TNFα over time (6-, 12-, and 24-hours post-infection). Each row represents a gene, and each column corresponds to a time point across different infection conditions. Red indicates upregulation, while green indicates downregulation compared to uninfected control cells.

In mono-viral infections, gene expression levels were consistently higher, indicating a stronger cellular response. In contrast, co-infections with GAS or *S. pneumoniae* showed more distinct gene changes, as showed in **Figure 4**. This suggest that bacterial co-infection may modulate or suppress the host’s response to viral infection.

The heatmap in **Figure 4**, reveals that both co-infection conditions follow a similar expression pattern, while the viral-only condition shows distinct upregulation. This supports that viral infection alone elicits a more dynamic and pronounced cellular response. Notably, *MUC5C* displayed a delayed but stronger upregulation compared to MUC2, indicating differential regulation of mucin genes during infection.

**Figure 5:**
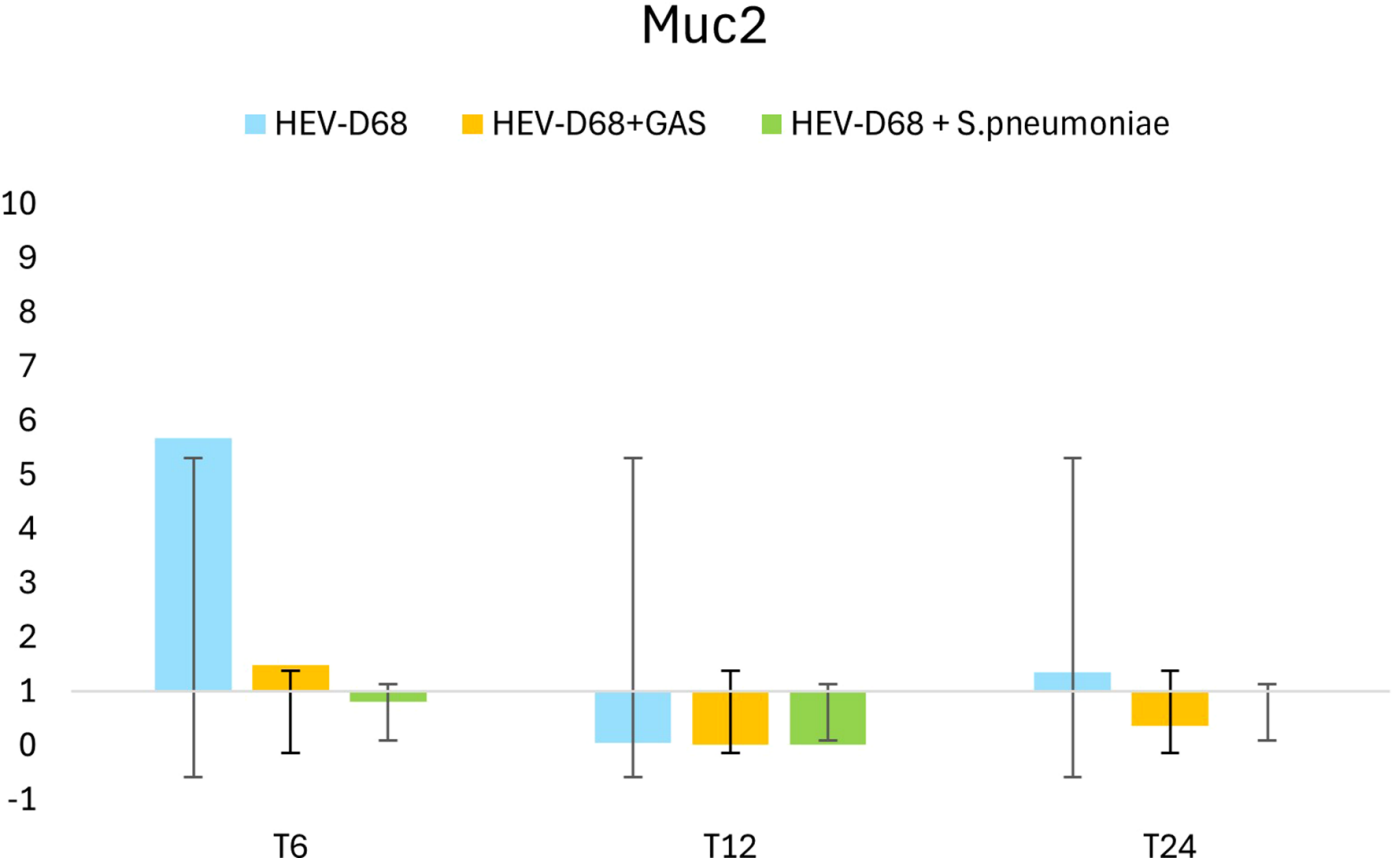
Shows the 2-fold change in relative expression of MUC2 in A549 cells across different infection conditions and time points. The left panel displays expression levels with standard deviation error bars, while the right panel illustrates the trend over 1 DPI. From the first measurement 6 hours post-infection (T6) when the infection pressure is continuous. At 6 hours the medium with the pathogen was removed and changed and measured every 6 hours until 24 hours.

During the first 6 hours, when infectious pressure was highest, MUC2 was upregulated in viral infections. After the medium was refreshed at T6, expression declined by 12 hours, suggesting reduced mucin production as the immediate viral threat subsided. By 24 hours, MUC2 expression increased slightly, possibly reflecting a secondary response to ongoing infection. Both *MUC2* and *MUC5AC* are secreted, gel-forming mucins (Sheng and Hasnain, 2022), and their expression is closely tied to pathogen presence in the environment. The initial upregulation corresponds to active pathogen interaction, while later changes reflect the host’s adaptive response as showed in **Figure 5**.

**Figure 6:**
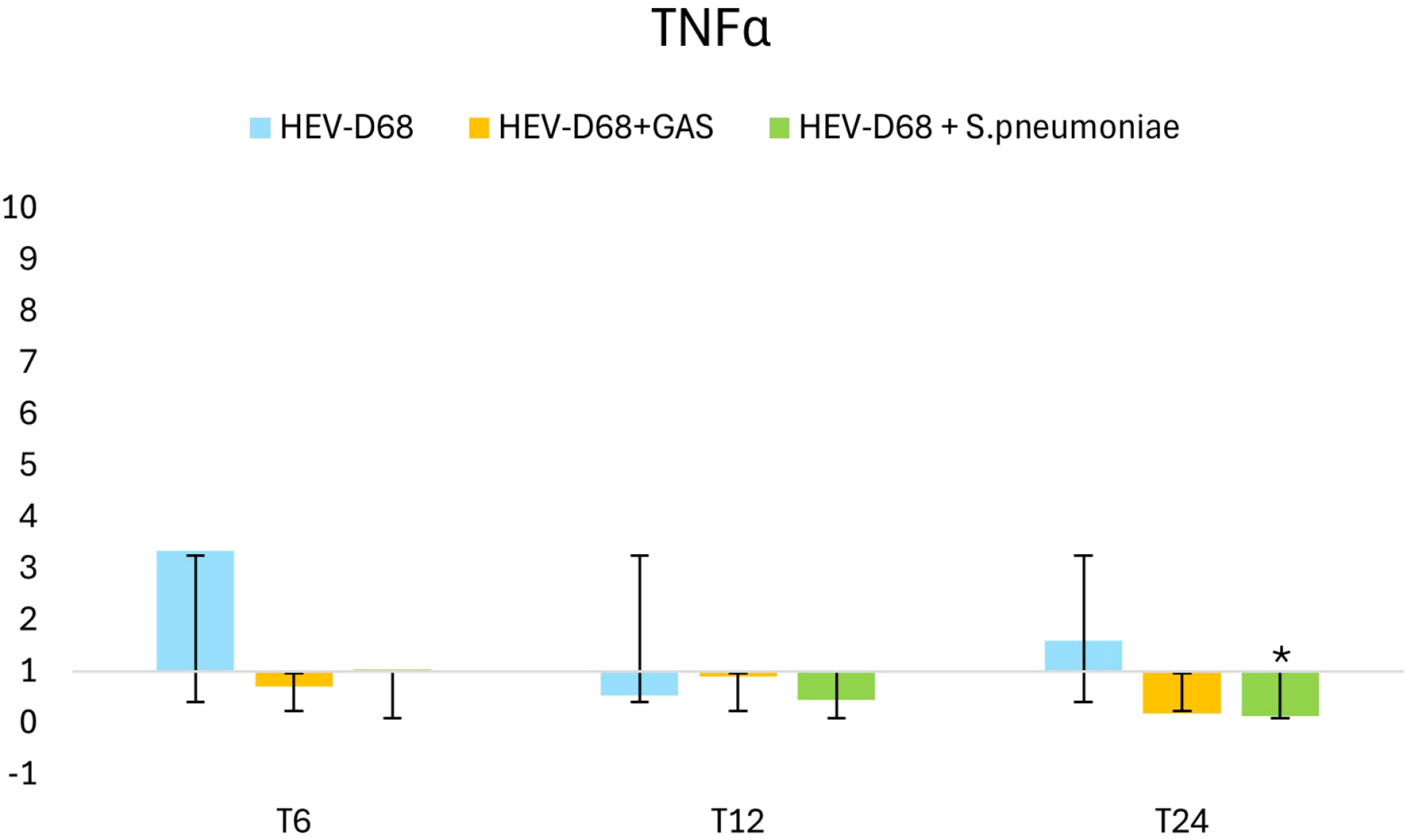
Shows 2-fold change in relative expression of TNFα in A549 cell. The left panel presents expression levels with standard deviation error bars, the significant changes (p < 0.05) are marked with an asterisk (*). The right panel illustrates expression trends over 1 DPI. From the first measurement 6 hours post-infection (T6) when the infection pressure is continuous. At 6 hours the medium with the pathogen was removed and changed and continuously measured every 6 hours until 24 hours.

TNFα expression peaked between 6- and 12-hours post-infection, coinciding with active viral replication and high infectious pressure as shown in **Figure 6**. After the medium was refreshed at T6, expression declined, indicating a reduced immune response as extracellular pathogen levels decreased. However, as intracellular replication continued, expression began to rise again toward 24 hours. In co-infections, *TNFα* expression was less pronounced, suggesting that bacterial presence may dampen the immune response triggered by the virus. This supports the observation that mono-viral infections elicit a stronger cellular reaction compared to co-infections.

### Sialic acid biosynthesis

The interaction between viruses and sialic acid receptors is believed to trigger the sialylation biosynthesis (Kim, 2020). One of the most common forms of sialic acid in humans is N-acetylneuraminic acid (Neu5Ac), which contributes to mucus formation in the respiratory epithelium by capping the ends of glycan chains in both N-glycosylation and O-glycosylation processes (Haines-Menges et al., 2015; Brockhausen et al., 2009; Traving and Schauer, 1998). O-glycosylation of mucins is a critical post-translational modification (PTM) required to ensure proper protein function (Tarp and Clausen, 2008).

Key enzymes involved in sialic acid biosynthesis include *CMAS*, *GNE* and *NANS* which represent different steps in the sialylation pathway (Lee et al., 2021). Additionally, Siae modifies sialic acid on the cell surface through O-acetylation, making it available for viral binding (Ide et al., 2024).

Despite the high initial infection pressure, only a small proportion of viruses are taken up by the cells in the early stages. The biosynthesis of sialic acid is triggered as a cellular response to infection, particularly when the availability of sialic acid receptors is depleted. After the initial exposure, viruses attach to or are internalized by cells begin replicating, releasing new virions that go on to infect neighboring cells. This cascade increases the demand for sialic acid biosynthesis.

**Figure 7:**
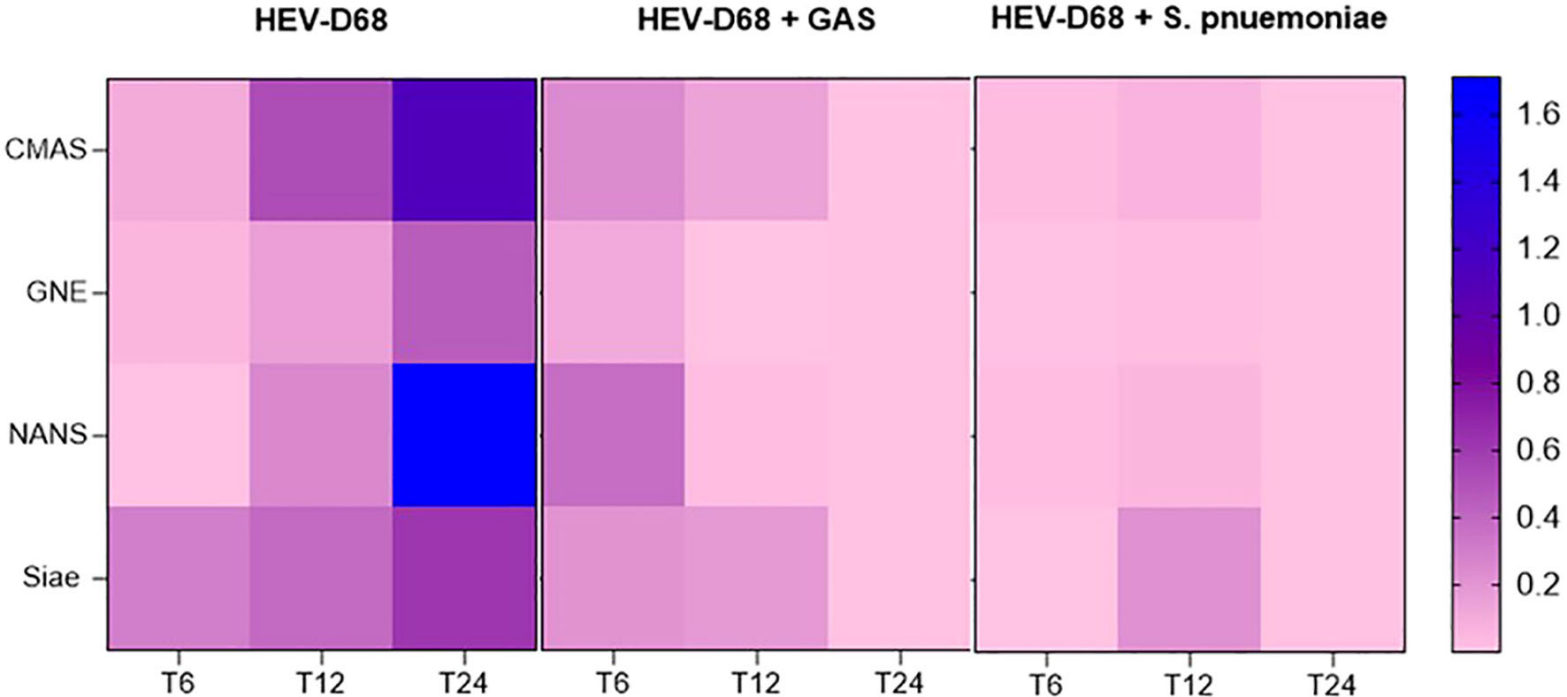
Presents a heatmap showing the relative expression of CMAS, GNE, and NANS over time (6-, 12-, and 24-hours post-infection). Each row represents a gene, and each column represents correspond to a time point across different infection conditions. Blue indicates upregulation, while pink indicates downregulation compared to uninfected control cells.

It is evident that *CMAS* and *NANS* are more dynamically regulated than *GNE*, particularly in mono-viral infections where both genes as upregulated, as shown in heatmap (**Figure 7***)*. In contract, all co-infection conditions show consistent downregulation across all four biomarkers, suggesting that bacterial presence may supress the sialylation response.

**Figure 8:**
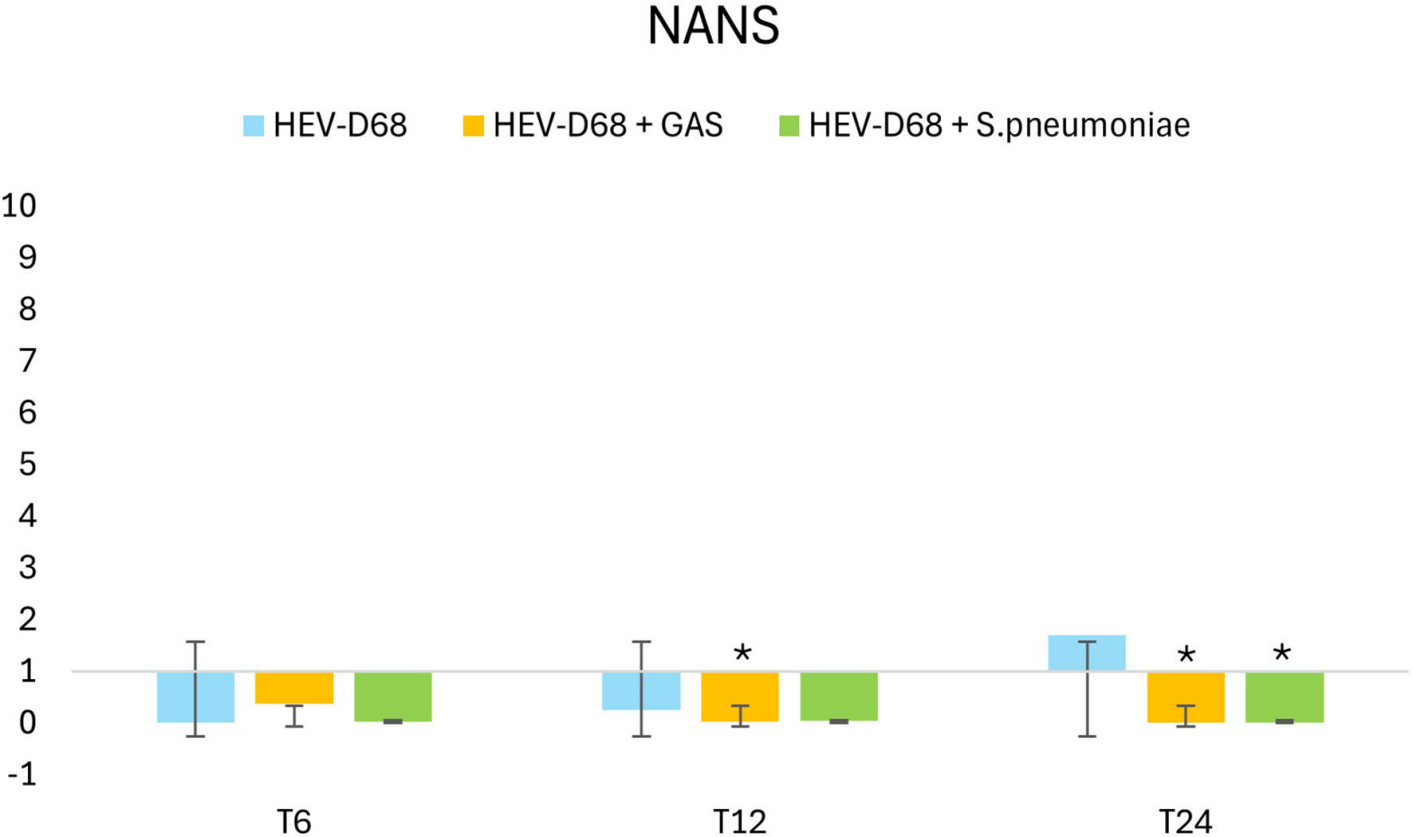
Shows 2-fold change in relative expression of NANS in A549 cells. The left panel expression levels with standard deviation error bars, and significant changes (p < 0.05) are marked with an asterisk (*). The right panel illustrates expression trends over 1 DPI. From the first measurement 6 hours post-infection (T6) when the infection pressure is continuous. At 6 hours the medium with pathogen was removed and changed and continuously measured every 6 hours until 24 hours.

**Figure 8** presents the relative gene expression of *NANS* that shows a downregulation for all the infections. At 6 hours (T6), viral attachment begins, and a small number of viruses are internalized. Between 6 and12 hours, viral uptake increase, initiating biosynthesis. By 24 hours, the expression of NANS rises further in mono-viral infections, reflecting the growing demand for sialic acid synthesis. However, in co-infections with GAS and S. pneumoniae, NANS expression is significantly downregulated, despite the presence of infectious pressure. This suggests that bacterial co-infection may interfere with og supress the host’s biosynthetic response.

Siae is the enzyme responsible for modifying sialic acid on the cell surface, making it accessible for viral binding. Interestingly, while viruses directly utilize sialic acid as a receptor, bacteria such as *S. pneumoniae* and GAS cleave the molecule but do not consume the receptor in the same way. In co-infection with *S. pneumoniae*, *Siae* expression is significantly downregulated after 6 hours (T6) when the infectious pressure is high. Still the gene expression of *Siae* in the co-infection is significantly downregulated after 24 hours. These findings highlight the distinct impact of bacterial co-infection on host cell biosynthetic pathways compared to viral infection alone.

## Discussion

In this study, we investigated the effect of viral infection compared to bacterial co-infection on epithelial host cells through comprehensive gene expression analysis. To examine the differential impact of bacterial co-infection with Group A Streptococcus (GAS) and *S. pneumoniae* on A549 lung epithelial cells compared to infection with human enterovirus D68 (HEV-D68) alone, we conducted an *In Vitro* experiment. The aim of this study is to gain a deeper understanding of the underlying mechanisms of viral infection and bacterial co-infection in lung cells, with the goal to develop more effective treatments in the future.

### Cell viability

Samples collected after 36 hours were excluded due to the high infection pressure, which rendered them unrepresentative. At this time point, approximately 50% of the cells has died. The remaining cells at 36 hours (T36) would consist of a mix of infected and non-infected cells, including both dead infected cells and viable non-infected cells. Since we measure the total gene expression based on an equal number of viable cells, the cell population at 36 hours would not accurately reflect the expression profile of pathogen-affected cells.

To extend the experiment duration, we could have reduced the infection pressure from the beginning. This would allow us to isolate RNA from a consistent proportion of viable cells across all samples. Theoretically, based on the viral replication cycle illustrated in **Figure 2**, the ratio of cells to virus at 36 hours would resemble that observed at 12 hours. A prolonged experiment with reduced infection pressure would likely result in a delayed infection process, with the time points such as 36 and 48 hours reflecting similar gene expression patterns to those seen at 12 and 24 hours.

### The cellular response to pathogen infection

The immune response is a coordinated process involving various components of the immune system working together to protect cells from external threats. It is evident that the cellular response is entirely different during co-infection, indicating that distinct processes are occurring in the cellular environment. When epithelial cells encounter extracellular pathogens, they initiate immune responses. Mucins, which are expressed in epithelial cells, play a crucial role in promoting cell survival and protecting against external stimuli. Mucus serves a dual purpose: it acts as a vital defense mechanism in the respiratory tract by trapping and clearing pathogens, while also being exploited by pathogens (Zanin et al., 2016).

Epithelial cells are known to produce mucus in response to viral infections such as influenza virus, RSV, and SARS-CoV-2 (Mettelman et al., 2022). This is also observed with HEV-D68, as shown in **Figure 3**. Mucins are a part of the cellular defense mechanism that prevents pathogens from anchoring to the cells. The upregulation of *MUC2* during viral infection is expected, as cells attempt to block pathogens attachment (Rose et al., 2001). The slight upregulation of *MUC2* may indicate a secondary response or a return to baseline levels as the cells adapt to the new environment. The trends for all the distinct infections show differences in gene expression.

HEV-D68 infection triggers a dynamic cellular response, as illustrated in **Figure 5***(MUC2).* During co-infections, however, changes in the surrounding environment alter the cellular response. The virus appears to affect cells differently when competing with bacteria, resulting in gene expression patterns that diverge from those seen in mono-viral infections. Bacterial virulence factors, such as pneumolysin from *S. pneumoniae* disrupt epithelial integrity and suppress antiviral responses by modulating host signaling pathways (Rai et al., 2016). This suppression may explain the reduced expression of mucin and cytokine genes during co-infections. Bacterial factors interfere with host defenses, leading to a more complex and potentially attenuated immune response compared to mono-viral infections (Fieber and Kovarik, 2014; Melvin and Bomberger, 2016).

This downregulation may indicate that the immune system is navigating a more complex regulatory environment. GAS and *S. pneumoniae* produce virulence factors that facilitate immune evasion, contributing to the downregulation of *TNFα* (Rahman and McFadden, 2006). The protein kinase *p38* MAPK is activated by various stimuli, including molecules like *TNFα*. *p38* is a key enzyme in intracellular signaling pathways involved in stress-induced production of IL-1β and *TNFα* (Bachstetter et al., 2011). Activation of stress-induced pathways such as *p38* MAPK may lead to barrier disruption and enhanced bacterial invasion.

### Sialic acid biosynthesis

The genes *GNE*, *NANS*, *CMAS* and *Siae* are integral components of the sialic acid biosynthesis pathway, which plays a crucial role in cell-cell interactions and immune responses. These biomarkers are part of the same pathway and are considered interdependent. Upregulation or downregulation of on gene can lead to compensatory changes in the expression of others to maintain balance in the sialic acid production (Awasthi et al., 2021). During viral infection, all enzymes in this pathway are expected to be upregulated in response to the need to restrain viral spread and maintain cellular functions. The upregulation was confirmed, as shown in **Figure 7**. *GNE*, the first enzyme in the biosynthesis pathway, is considered the “master regulator”. Its upregulation during viral infection is expected, as cells must ensure the availability of ManNAc-6- for subsequent steps in the pathway (Bhide and Colley, 2017). The stronger upregulation of *NANS* may reflect increased demand for Neu5Ac during viral infection. The difference in upregulation between *GNE* and *NANS* can be explained by their respective roles in maintaining the flow and output of the pathway (Bhide and Colley, 2017). *CMAS* upregulation is also triggered by the increased demand for sialic acid. *CMAS* activates free sialic acids by transferring cytidine monophosphate (CMP) from cytosine triphosphate (CTP) to the hydroxyl group at C2 - a process that initiates sialylation, where sialic acid is added to glycolipids and glycoproteins (Bhide and Colley, 2017).

**Figure 9:**
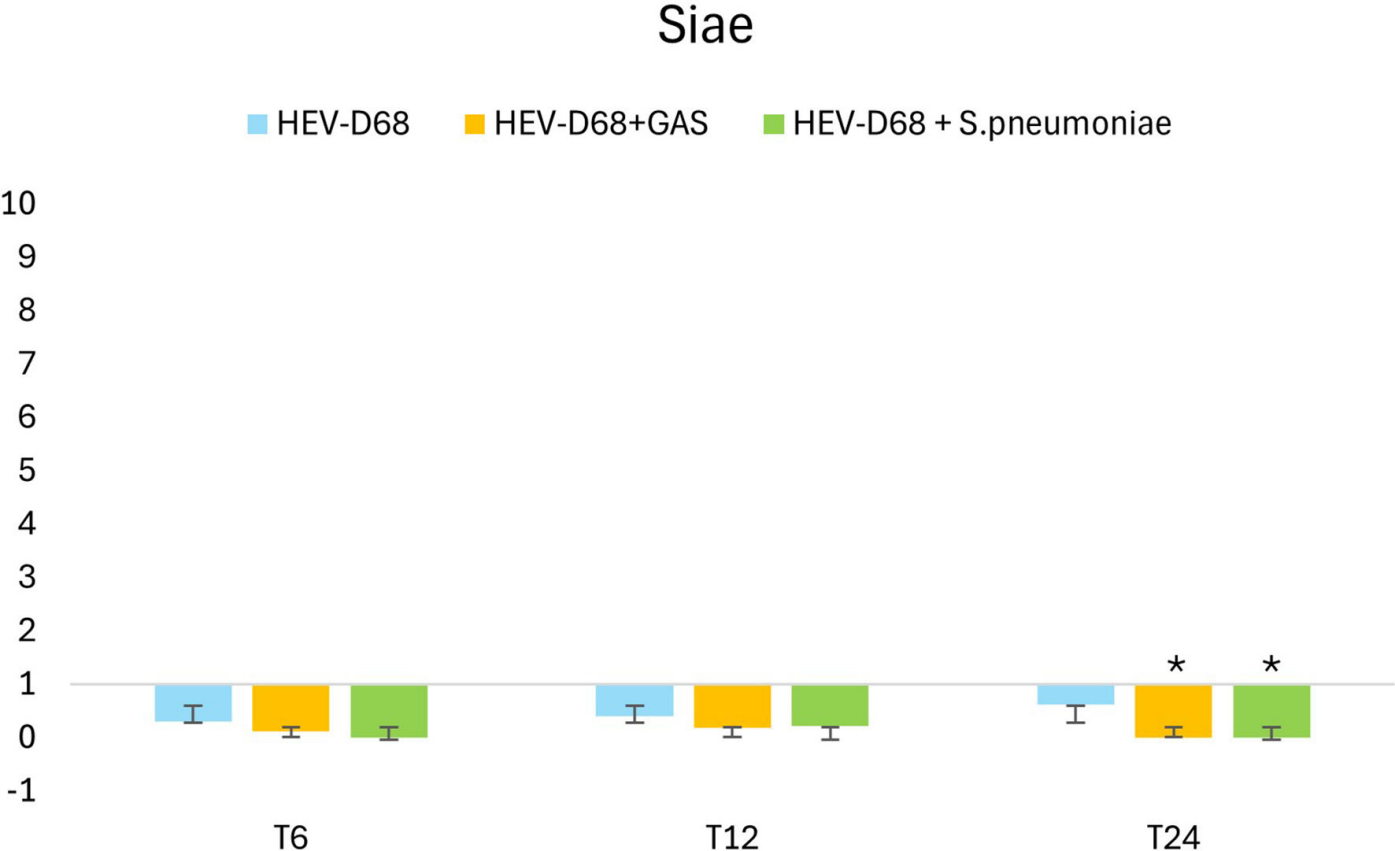
Shows the 2-fold change in relative expression of Siae in A549 cells. The left panel expression levels with standard deviation error bars. Significant changes are marked with an asterisk (*) for p < 0.05 and double asterisk (**) for p < 0.01. The right panel illustrates expression trends over 1 DPI. From the first measurement 6 hours post-infection (T6) when the medium with pathogen was removed and changed. Through every 6 hours until 24 hours.

The major differences in infections lie in how efficiently pathogens bind and anchor to host-cell receptor. In co-infection with GAS, all genes show slight upregulation to meet the increased cellular demand for more sialic acid. However, despite the presence of virus - which typically triggers downstream gene expression - the expected changes do not follow the infection’s progression. For both co-infections, gene expression does not align with the anticipated development of infection. Interestingly, in co-infection with *S. pneumoniae*, there is no early response or change in gene expression, implying that *S. pneumoniae* exerts an immediate effect on host cells. Both GAS and *S. pneumonia* have evolved strategies to interfere with host cell signaling (Fieber and Kovarik, 2014; Melvin and Bomberger, 2016). The presence of bacterial pathogens may alter the host’s gene expression profile, suppressing the demand for sialic acid production and impacting the progression of the viral infection (Melvin and Bomberger, 2016). The bacterial impact in co-infection appears to be more pronounced that the typical demand triggered by mono-viral infection. While mono-viral infection stimulates cells to produce more sialic acid, co-infections do not. One possible explanation is that pathogens compete for receptors on the cell surface, which are not consumed, reducing the need for turnover and production of new sialic acid molecules – resulting in reduced gene expression, as shown in **Figure 9**. Bacteria compete with viruses for species-specific resources, nutrients, receptors and cell viability.

The variability observed between mono-viral infection and bacterial co-infection can be attributed to difference in pathogen replication rates. *S. pneumoniae* has a generation time of approximately 24–36 under optimal conditions, whereas HEV-D68 has a replication cycle of about 8–12 hours. This cycle includes viral entry, replication, assembly of new particles, and release (Wang et al., 2017). These differences highlight the faster reproduction rate of viruses compared to bacteria. Viral replication is influenced by host cell conditions, while bacterial growth depends on nutrient availability and the microenvironment shaped by viral infection. Virus-induced cell lysis releases nutrients, which can enhance bacterial proliferation. Additionally, changes in pH and oxygen levels caused by viral infection may affect bacterial growth. To facilitate the interpretation, the genes are categorized into two groups: cellular response and sialic acid biosynthesis.

### Overview and future prospects

The primary goal of this experiment was to investigate the lung epithelial cellular response to infection. Our findings reveal that the cellular response differs between mono-viral infections and bacterial co-infections. This variation is likely linked to the virus’s mechanism of binding to and consuming sialic acid receptors, which in turn triggers the sialylation biosynthesis pathway. While our study provides valuable preliminary insights, further research is necessary to confirm the specific impact of HEV-D68 and bacterial co-infection on epithelial cells. HEV-D68 may interact with host cells in ways that differ from other respiratory viruses, and these unique interactions warrant deeper investigation. The use of A549 cells offers a practical model for studying respiratory epithelial responses; however, it comes with limitations. A549 cells may not fully replicate the receptor expression profiles or immune responses of primary lung epithelial cells. This could influence the interpretation of our results, particularly regarding receptor-mediated interactions and immune signaling. Future studies should incorporate primary lung and airway epithelial cells to validate these findings and better assess the microenvironment created during infection.

Another important consideration is the influence of the infection on the growth environment for both virus and bacteria. Viral replication and bacterial proliferation are shaped by the microenvironment, including nutrient availability, pH, oxygen levels, and cell viability. A more detailed understanding of how these factors interact and evolve during co-infection could provide critical insights into pathogen dynamics and host responses.

To advance this research, future studies should focus on validating findings in primary epithelial cell models to ensure physiological relevance and investigate the competitive interactions between viruses and bacteria for cellular receptors and nutrients. By targeting key points in interaction – such as receptor binding, immune evasion, and nutrient acquisition – novel therapeutic strategies could be developed. These may include blocking viral attachment and entry, inhibiting bacterial nutrient uptake, or modulating host cell signaling pathways to enhance immune defense. Such approaches could significantly reduce the severity of respiratory infections and improve treatment outcomes.

## Data Availability

The datasets presented in this study can be found in online repositories. The names of the repository/repositories and accession number(s) can be found in the article/supplementary material.
